# Linked Supramolecular Building Blocks for Enhanced Cluster Formation

**DOI:** 10.1002/chem.201405746

**Published:** 2015-01-09

**Authors:** Ross McLellan, Maria A Palacios, Christine M Beavers, Simon J Teat, Stergios Piligkos, Euan K Brechin, Scott J Dalgarno

**Affiliations:** [a] Institute of Chemical Sciences, Heriot–Watt University Riccarton, Edinburgh, Scotland EH14 4AS (UK) E-mail: S.J.Dalgarno@hw.ac.uk; [b] School of Chemistry, University of Edinburgh David Brewster Road, Edinburgh, Scotland EH9 3FJ (UK) E-mail: ebrechin@staffmail.ed.ac.uk; [c] Station 11.3.1, Advanced Light Source, Lawrence Berkeley National Laboratory 1 Cyclotron Road, Berkeley, CA94720 (USA); [d] Department of Chemistry, University of Copenhagen Universitetsparken 5, 2100 (Denmark) E-mail: piligkos@kiku.dk

**Keywords:** calixarenes, clusters, coordination chemistry, magnetism, supramolecular chemistry

## Abstract

Methylene-bridged calix[4]arenes have emerged as extremely versatile ligand supports in the formation of new polymetallic clusters possessing fascinating magnetic properties. Metal ion binding rules established for this building block allow one to partially rationalise the complex assembly process. The ability to covalently link calix[4]arenes at the methylene bridge provides significantly improved control over the introduction of different metal centres to resulting cluster motifs. Clusters assembled from bis-calix[4]arenes and transition metal ions or 3*d*-4*f* combinations display characteristic features of the analogous calix[4]arene supported clusters, thereby demonstrating an enhanced and rational approach towards the targeted synthesis of complex and challenging structures.

## Introduction

**Figure 1:**
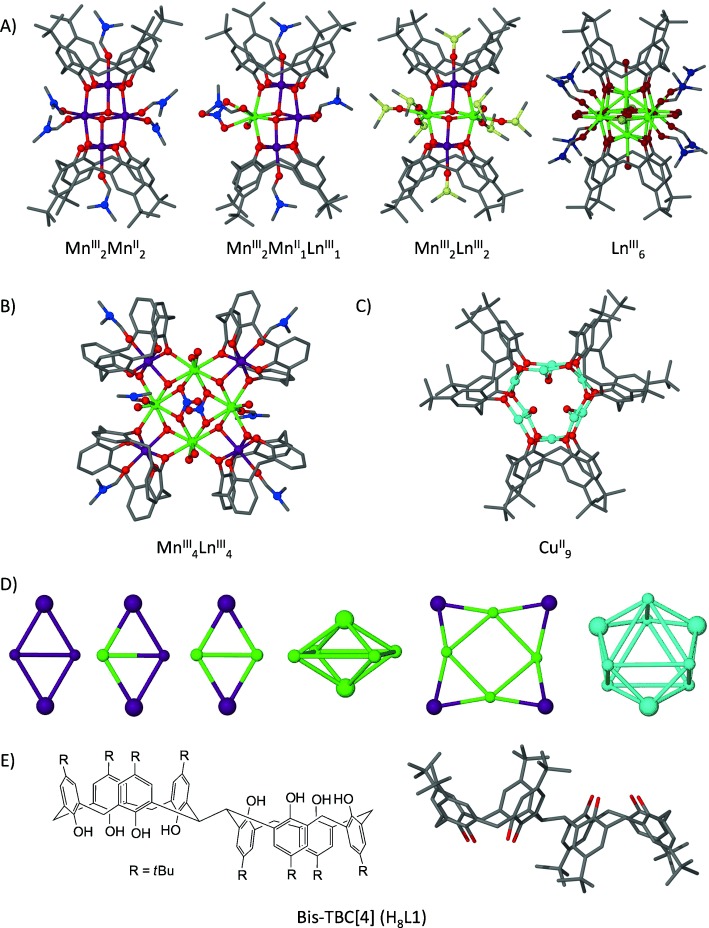
Selected polymetallic TBC[4]-supported clusters and the related bridge-linked bis-TBC[4]. A) Clusters supported by two TBC[4]s with tailored metal composition (Mn_4_, Mn_3_Ln_1_, Mn_2_Ln_2_ and Ln_6_).[4, 5, 9, 12] B) Square within square Mn^III^_4_LnM^III^_4_ clusters supported by four TBC[4]s.[6, 7] C) Tricapped trigonal prismatic Cu_9_ cluster motif with anions and ligated solvent omitted for clarity.[10] D) Metallic skeletons of clusters shown in A–C with capping TBC[4]-TM/LnM moieties drawn as large spheres. E) Two views of bis-TBC[4] showing the antiparallel arrangement and hydrogen-bonding interactions at the TBC[4] lower-rims. Colour code: Mn, purple; Ln, green; Cu, pale blue; N, royal blue; C, grey; O, red; S, yellow; H atoms are omitted for clarity.

Many strategies have been employed in the synthesis of polymetallic clusters of paramagnetic metal ions. These range from serendipitous assembly through to rational design, the latter of which naturally relies on targeted ligand composition. The ultimate goal is the same in all cases, that being the isolation of molecules that are of interest to chemists, physicists and theoreticians due to the prevailing magnetic properties. Predicting the assembly of such clusters from multi-component systems, especially from a viewpoint of tailoring their magnetic properties, represents a significant challenge to the synthetic coordination chemist.[1, 2] *p*-*t*Bu-calix[4]arene (TBC[4]) has emerged as a highly versatile building block for the construction of polynuclear transition metal (TM), lanthanide metal (LnM) and 3*d*-4*f* clusters.[3–10] Its polyphenolic character allows it to: 1) bind, and 2) bridge to metal centres within clusters. We have used TBC[4] to form a library of clusters in which TM/LnM-TBC[4] moieties act as capping vertices (Figure 1 A–D). Our first significant development was isolation of a family of Mn^III^_2_Mn^II^_2_(TBC[4])_2_ single-molecule magnets (SMMs; Figure 1 A).[4, 5] The central polymetallic core is an unusual butterfly in that the oxidation states are reversed relative to those typically observed with other ligands.[11] This occurs because the Jahn–Teller distorted, axially elongated Mn^III^ ions are preferentially bound in the TBC[4] pocket; TBC[4] provides a stable coordination environment for a metal ion that can accommodate four short equatorial and two long axial bonds. The TBC[4] O atoms bridge to the centrally located Mn^II^ ions and the structure can be viewed rather simply as two [Mn^III^(TBC[4])(OH)(DMF)] metalloligands encapsulating two “naked” Mn^II^ ions, the remaining coordination sites of which are filled with solvent. The concept that two or more Mn^III^ metalloligands can encapsulate other metal ions in a centrally located pocket therefore suggests that a variety of homo- and heterometallic cages with similar structures should be isolable. Indeed we recently found it possible to tailor the butterfly composition so as to systematically incorporate LnM^III^ ions in place of one or both Mn^II^ ions within Mn_*x*_LnM_*y*_TBC[4]_2_ clusters (where *x*=4/*y*=0, *x*=3/*y*=1 and *x*=2/*y*=2), thereby representing unprecedented structural control.[12] Analogous reactions with LnM^III^ ions in the absence of TM^II^ or TM^III^ ions affords LnM^III^_6_TBC[4]_2_ octahedra (Figure 1 A).[9] In addition we have synthesised a series of square-within-square 3*d*-4*f* clusters (Mn^III^_4_LnM^III^_4_TBC[4]_4_, LnM=Gd, Tb or Dy) in which four [Mn^III^(TBC[4])(OH)(DMF)] metalloligands encapsulate four “naked” Ln^III^ ions. These behave as SMMs or cryogenic magnetic refrigerants depending on the LnM employed (Figure 1 B).[6, 7] Finally, treatment of TBC[4] with Cu^II^ salts affords enneanuclear clusters that display versatile anion binding capabilities.[10] These display an interesting structural departure in that the polyphenolic pocket binds Cu^II^ ions to form moieties that cap a Cu^II^ trigonal prism (Figure 1 C). From these key developments we have established metal ion binding rules for TBC[4]. We have found that under ambient conditions TBC[4]: 1) preferentially binds Mn^III^ ions, 2) will bind TM^II^ ions (e.g., Cu^II^ and Co^II^) in the absence of TM^III^ ions (e.g., Mn^III^), and 3) will bind LnM^III^ ions in the absence of TM^II^ or TM^III^ ions.

A range of molecules comprising TBC[4]s modified at the methylene bridge were recently reported in the literature, including linked C[4]s.[13] This presented an opportunity to build on the TBC[4] binding rules by having two metal ion complexation sites within bis-TBC[4] (Figure 1 E). We therefore predicted that, upon structural rearrangement of the ligand, it would be possible to construct: 1) Mn^III^/Mn^II^ clusters comprising more than two [Mn^III^-TBC[4]]^−^ moieties, 2) LnM_*x*_ clusters comprising more than two [LnM^III^-TBC[4]]^−^ moieties (in the absence of TM^II^/TM^III^ ions), 3) 3*d*-4*f* clusters containing more than two [Mn^III^-TBC[4]]^−^ moieties and LnM^III^ ions from Mn/LnM combinations, and 4) polynuclear Cu^II^ clusters containing more than two [Cu^II^-TBC[4]]^2−^ moieties (in the absence of other TM^II^/TM^III^ ions). In the first of our experiments we have achieved three of these four objectives, and the resulting clusters display predicted structural characteristics within a series of spectacular new motifs, all of which demonstrate a significant step towards the targeted assembly of polymetallic species. Bis-TBC[4] (H_8_L1) was synthesised according to literature procedure,[13] and upon crystallisation was found to adopt an up-down twisted arrangement (Figure 1 E). The TBC[4] cavities are stabilised by lower-rim H bonding, point in alternate directions, and neighbouring molecules assemble to form an antiparallel bilayer (Figure S1 in the Supporting Information) reminiscent of that observed for TBC[4]. The conformational versatility of calixarenes is generally well understood; the CH_2_ bridges between phenyl rings impart flexibility that allows ring inversion to proceed in the solution phase with very low accompanying energy barriers.[14] Extension of this to H_8_L1 allows for the possibility that two sets of TBC[4] phenolic oxygen atoms may, upon rotation/inversion, orient such that they are directed towards a central point. Furthermore, the proximity of two phenolic oxygen atoms from each TBC[4] fragment would present additional binding sites for metal ions. In particular we anticipated that H_8_L1 would allow us to controllably introduce multiple metal centres of predetermined oxidation states (e.g., two [Mn^III^-TBC[4]]^−^ moieties) in order to enhance the formation of new polynuclear metal clusters.

## Results and Discussion

**Figure 2:**
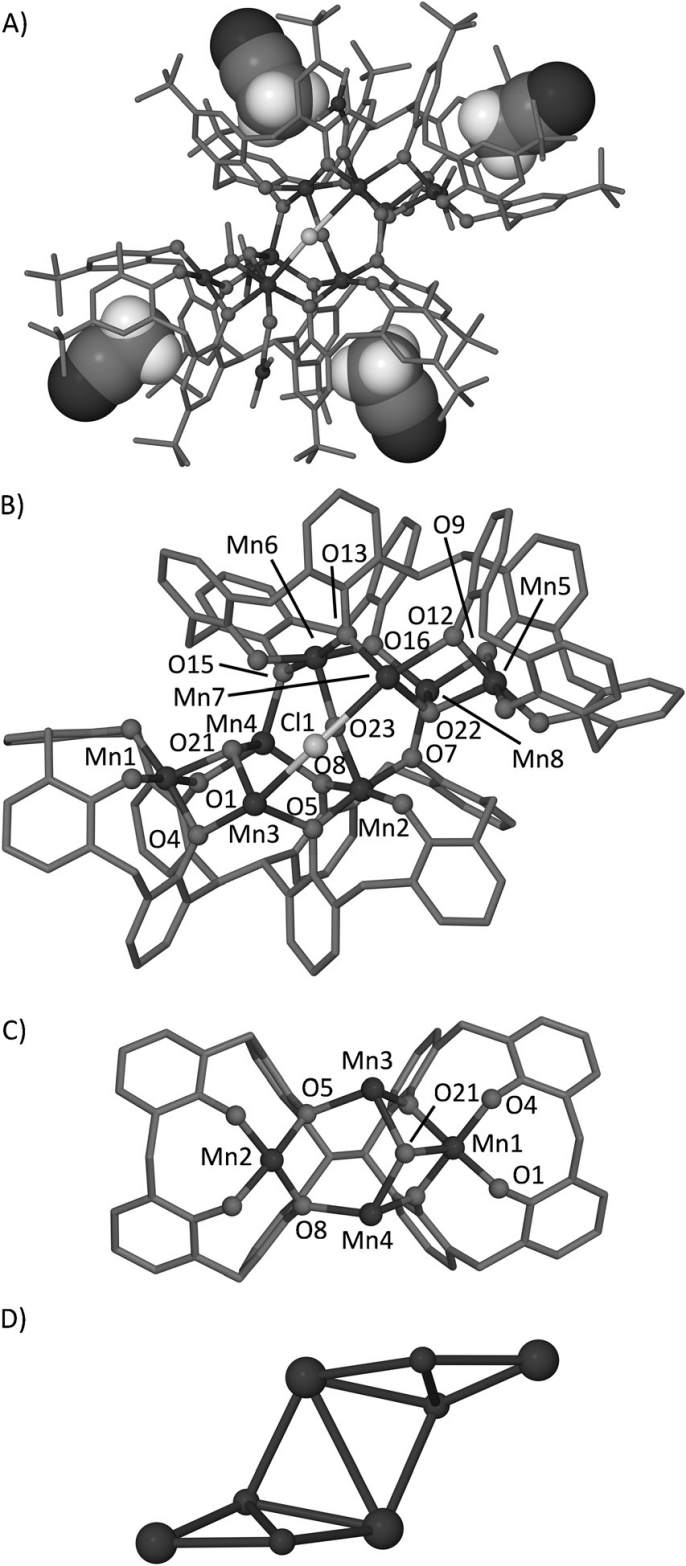
Views of the single-crystal X-ray structure of 1 showing the polymetallic Mn^III^_4_Mn^II^_4_ core. A) Cluster in 1 showing acetonitrile molecules in space-filling representation occupying the bis-TBC[4] cavities. B) Polymetallic core of 1 showing the two butterfly units linked through phenolate, chloride and hydroxide bridging. C) Detailed view of one butterfly within 1. D) Metallic skeleton of 1 with the capping [TBC[4]-Mn^III^] moieties drawn as large spheres. H atoms and non-coordinating solvent (other than cavity bound MeCN in A) are omitted in A–C. Ligated solvent omitted in B and C.

Reaction of H_8_L1 with manganese(II) chloride hydrate in a DMF/MeOH mixture and in the presence of base afforded single crystals of [Mn^III^_4_Mn^II^_4_(L1)_2_(μ_3_-OH)_2_(μ-OH)(μ-Cl) (H_2_O) (MeOH)(dmf)_4_]⋅2 H_2_O⋅12 MeCN (**1**), following slow diffusion with acetonitrile (Figure 2).[15] The isolated complex contains a mixed valence Mn^III^_4_Mn^II^_4_ cluster that is strikingly similar to the archetypal Mn^III^_2_Mn^II^_2_ butterfly motif isolated with TBC[4].[4, 5] Both L1 ligands in **1** are arranged such that one TBC[4] fragment has undergone inversion, presumably as a consequence of metal ion coordination. In this conformation L1 is ideally suited to controllably insert two Mn^III^ ions into a cluster. Furthermore, additional binding sites between the TBC[4] fragments in each L1 are occupied by Mn^II^ ions. The structure of **1** is best described as two L1-supported distorted Mn^III^_2_Mn^II^_2_ butterflies, or alternatively as two Mn^III^-TBC[4] metalloligands encapsulating two “naked” Mn^II^ ions. The metal ions are linked together by bridging phenolates, hydroxide and chloride (or formate[15] generated by in situ hydrolysis of dmf). The metal core and coordination environment relating to each “Mn_4_” (Mn1–Mn4 cf. Mn5–Mn8) is essentially the same, and so only one half of the metal cluster will be described in detail. Mn1 is centrally bound to four fully deprotonated phenolic oxygen atoms O1–O4 (Mn–O range 1.865(3)–1.956(3) Å) of one TBC[4] moiety. As predicted the square pyramidal Mn1 is in the third oxidation state and its coordination is completed by bonding to a μ_3_-hydroxide (Mn1–O21 2.126(3) Å), which is further bonded to Mn3 and Mn4 (2.152(3) and 2.155(3) Å, respectively), both of which are in the second oxidation state; Mn3 lies in the binding pocket generated by inversion. In addition to O21, Mn3 is bonded to dmf (Mn3–O17 2.104(4) Å) and two μ-phenoxide oxygen atoms (Mn3–O4 2.266(3) Å and Mn3–O5 2.175(3) Å). The remaining two coordination sites display extensive disorder. The first component describes a distorted octahedral metal ion bonded to a half occupancy μ-Cl^−^ (Mn3–Cl1 2.800(5) Å) that also connects to Mn7, and a MeOH/H_2_O ligand that each have quarter occupancy (Mn3–O24 2.196(5) Å).[16] Mn4 occupies the remaining binding cavity within L1. It is square pyramidal and is coordinated to, alongside O21, one dmf ligand (Mn4–O18 2.121(3) Å) and three μ-phenoxide oxygen atoms (Mn4–O1 2.208(2) Å, Mn4–O8 2.102(3) Å and Mn4–O15 2.129(3) Å). Mn2 is centrally bound to all four fully deprotonated phenolic oxygen atoms (O5–O8) of the second TBC[4] fragment of L1 (Mn–O range 1.902(3)–1.967(3) Å). It is also in the third oxidation state and possesses square pyramidal geometry. Mn2 is further bonded to a μ-hydroxide (Mn2–O23 2.067(3) Å), which bridges to the other similar “Mn_4_ cluster” within **1** (containing Mn5–Mn8).

Examination of the extended structure reveals that molecules of **1** pack in a complex fashion with adjacent clusters being well isolated (closest Mn⋅⋅⋅Mn distance ca. 13.1 Å). Along one direction, molecules assemble into an infinite chain such that two molecules of **1** lie perpendicular to, and sandwiched between, two adjacent molecules (Figure S2A in the Supporting Information). Viewing this arrangement from a perpendicular direction reveals that the four molecules assemble around a central solvent-filled channel (Figure S2B in the Supporting Information). Further packing reveals the formation of a tube-like structure (Figure S2C). While Mn_8_ clusters are not rare (a Cambridge Structural Database [CSD] search reveals about 65 entries) it is clear that the topology of the metal core in **1** is novel and a consequence of the L1 ligand.

**Figure 3:**
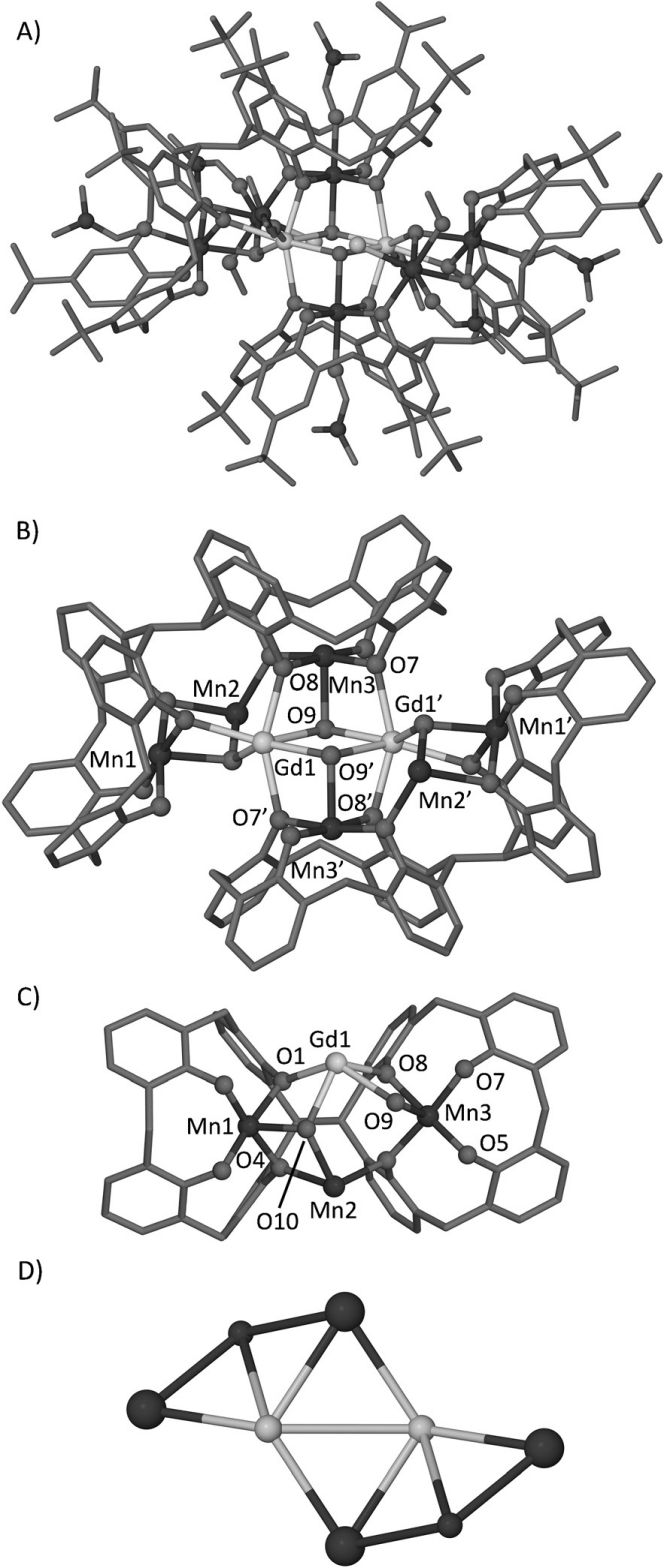
Views of the single crystal X-ray structure of 2 showing the mixed-valence polymetallic core. A) Cluster in 2 showing ligated dmf within each TBC[4] cavity, chloride anions and ligated MeOH. B) Polymetallic core showing the central Mn^III^_2_Gd^III^_2_ butterfly motif. C) Part of the asymmetric unit in 2 showing the distorted Mn^III^_2_Mn^II^Gd^III^ butterfly. D) Metallic skeleton of 2 with the capping TBC[4]-Mn^III^ moieties drawn as large spheres. H atoms and non-coordinating solvent are omitted in A–C. Ligated solvent and chloride anions are omitted in B and C.

Treatment of H_8_L1 with manganese(II) chloride and gadolinium(III) chloride in a DMF/MeOH mixture in the presence of base afforded single crystals of formula [Mn^III^_4_Mn^II^_2_Gd^III^_2_(L1-8 H)_2_(Cl)_2_(μ_3_-OH)_4_(MeOH)_2_(dmf)_8_]⋅(Et_2_O)_5_(dmf) (**2**) upon slow diffusion with diethyl ether (Figure 3). Structural analysis reveals, as predicted, that each TBC[4] fragment contains an Mn^III^ ion. As in **1**, the L1 ligands have undergone inversion, leading once more to additional binding pockets for metal ions. The metal core of **2** bears a remarkable similarity to **1**, the main difference being replacement of one Mn^II^ for one Gd^III^ in the binding site located between the two TBC[4] cavities of each L1. The central core is best described as a central Mn^III^_2_Gd^III^_2_ butterfly motif that is edge fused (sharing one Mn^III^ ion and one Gd^III^ ion) to two symmetry equivalent (s.e.) peripheral Mn^III^_2_Mn^II^Gd^III^ butterflies; Mn^III^-TBC[4] moieties are again found to encapsulate “naked” Mn^II^ and Gd^III^ ions. The central butterfly contains two s.e. distorted octahedral Mn^III^ ions (Mn3 and Mn3′) that each reside within a TBC[4] pocket. The coordination sphere of Mn3 consists of four phenolic oxygen atoms (one terminal and three bridging, with Mn3–O ranging from 1.872(4)–1.992(4) Å), a ligated dmf (Mn3–O11 2.224 Å) and a μ_3_-hydroxide (Mn3–O9 2.162(4) Å). The O(dmf)-Mn3-O(μ_3_-OH^−^) vector defines the Jahn–Teller axes with significant deviation from linearity (164.7(2)°). The central Gd^III^ ions (Gd1 and s.e.) are located in the binding sites situated between the two TBC[4] fragments of each L1 and are found to be seven coordinate (distorted pentagonal bipyramidal geometries). Each is bonded to the aforementioned μ_3_-hydroxide, O9, its s.e. (Gd1–O9 2.381(4) Å and Gd1–O9′ 2.362(4) Å, respectively), and to a second μ_3_-hydroxide, O10 (Gd1–O10, 2.328(4) Å). These are also coordinated to a ligated dmf and three μ-phenolic oxygen atoms (Gd1–O1 2.425(4) Å, Gd1–O8 2.298(4) Å and Gd1–O7′ 2.344(4) Å). Mn2 also resides in the cavity generated by the two TBC[4] fragments and has a distorted octahedral geometry. It is coordinated to a chloride ion (Mn2–Cl1 2.470(2) Å), two μ-phenolic oxygen atoms (Mn2–O4 and Mn2–O5, 2.342(4) and 2.212(4) Å, respectively), a μ_3_-hydroxide (Mn2–O10 2.165(4) Å), ligated dmf (Mn2–O14 2.096(4) Å) and ligated MeOH (Mn2–O15 2.271(4) Å). Mn1 (and s.e.) is coordinated centrally in the binding cavity of one TBC[4] fragment of L1 and has Mn1-phenolic oxygen lengths in the range 1.883(4)–1.976(4) Å. Two of these oxygen atoms (O1 and O4) bridge to Mn2 and Gd1, vide supra. Along the Jahn–Teller axis, Mn1 is coordinated to a μ_3_-hydroxide (Mn1–O10 2.195(4) Å) and a terminally bound dmf (Mn1–O12 2.243(4) Å) in the TBC[4] cavity. Adjacent clusters of **2** pack in a simple head-to-head zig–zag fashion (Figure S3 in the Supporting Information), each cluster being well isolated with closest distances of about 14.0 Å. A CSD search was performed to assess the structural novelty of **2**. Three entries were returned for Mn_6_Gd_2_ species, each containing a different topology from **2**. A second more general search for Mn_6_Ln_2_ was performed and this returned only eight entries, again all with different topologies.

**Figure 4:**
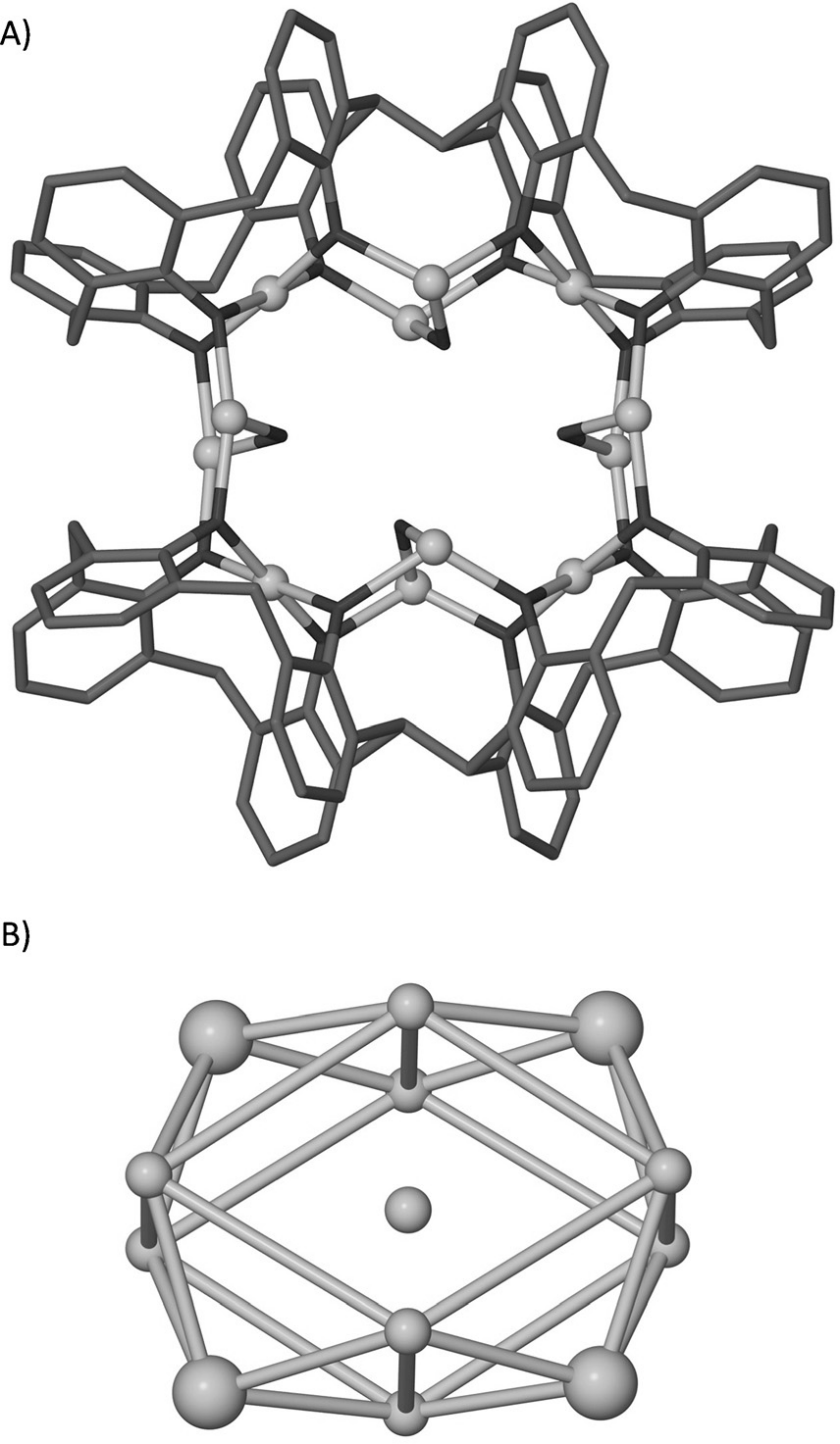
Views of the single-crystal X-ray structure of the cation in 3 showing the polymetallic core. A) Crown-like arrangement of outer twelve Cu^II^ centres showing bridging hydroxides on the interior. B) Metallic skeleton of the cation in 3 with the capping TBC[4]-Cu^II^ moieties drawn as large spheres. H atoms, OH/NO_3_ anions, ligated and non-coordinating solvent are omitted.

Treatment of copper(II) nitrate hydrate with H_8_L1 in a basic medium of DMF/MeOH afforded [Cu^II^_13_(L1)_2_(NO_3_)(μ-OH)_8_(dmf)_7_]⋅ (OH)(MeCN)_14_ (**3**) following diffusion of acetonitrile (Figure 4). Structure analysis reveals a Cu^II^_13_ cluster supported by two inverted L1 ligands. The TBC[4] pockets and additional binding sites are all occupied by Cu^II^ ions (Figure 4 A). The two Cu_4_L1 moieties are connected by four additional Cu^II^ ions (Figure 4 B), giving a polymetallic core that conforms to a tetracapped diamond (or tetracapped square prism). Each face is thus capped by a TBC[4]-Cu^II^ moiety, with the overall framework bearing obvious similarity to the tricapped trigonal prismatic Cu^II^_9_ clusters isolated from analogous reactions with TBC[4] (Figure 1 C). That is, the latter describes three Cu^II^-TBC[4] metalloligands encapsulating a trigonal prism, and the former describes four Cu^II^-TBC[4] metalloligands encapsulating a square prism. The remaining Cu^II^ ion in **3** resides in the centre of the prism and is disordered over several positions. The four capping Cu^II^ ions are in distorted square planar geometries and bond to all four phenolic oxygen atoms of each TBC[4] subunit (Cu–O range 1.926(5)–1.966(5) Å). Four distorted square planar Cu^II^ ions connect the two L1 ligands in **3** via O–Cu–O bridges (Cu–O range 1.914(5)–1.976(14) Å). Furthermore, each pair of bridging Cu^II^ ions is linked by a μ-OH (Cu–O range 1.914(5)–1.932(4) Å). Coordination is completed by bonding to either dmf or a nitrate ligand. The four Cu^II^ ions that reside in the binding sites between each TBC[4] fragment of L1 are either distorted trigonal bipyramidal or distorted tetrahedral, a fact largely dependent on the location of the final extensively disordered Cu^II^ ion. These five Cu^II^ ions are interconnected by a combination of five μ-OH ligands giving a cationic cluster.

The TBC[4]_3_Cu^II^_9_ cluster has been found to be a versatile anion binding material, with the core adapting such that three μ-hydroxides can be replaced by a μ_6_-carbonate, for example. The outer Cu^II^_12_ skeleton of **3** may thus potentially bind a range of different cations (organic or inorganic in nature), and this will be explored in detail in future studies. Analysis of the extended structure reveals that the assembly of **3** is best described as offset linear chains (Figure S4 in the Supporting Information). As expected, the metallic core in **3** is well isolated from adjacent clusters with a closest Cu⋅⋅⋅Cu distance of about 13 Å. A CSD search was performed to determine the novelty of the metallic skeleton in **3**. This returned one entry in which a Cu^I^_13_ cluster conforms to a pinwheel-like structure that is markedly different to that found in **3**.[17]

**Figure 5:**
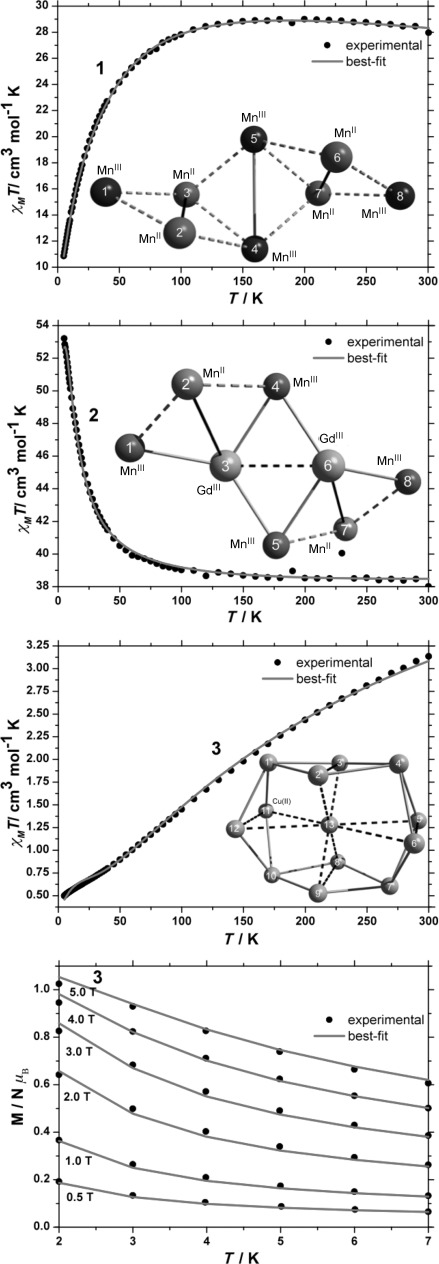
Experimental and best-fit *χ*_M_*T* products of 1–3 in a magnetic field of 0.1 T in the temperature range 5–300 K, and low-temperature magnetisation measurements on 3 in the temperature range 2–7 K in applied magnetic fields of 0.5, 1.0, 2.0, 3.0, 4.0 and 5.0 T. The inserts of the *χ*_M_*T* product plots represent the pair-connectivity of the various isotropic exchange interactions taken into account in the spin-Hamiltonian models for 1–3 for each metallic skeleton.

Direct current (d.c.) magnetic susceptibility studies were performed on polycrystalline samples of **1**–**3** in the temperature range 5–300 K in an applied magnetic field of 0.1 T. The results are shown in Figure 5 in the form of *χ*_M_*T* products, where *χ*=*M*/*B*, *M* is the magnetisation, *B* the applied magnetic field, and *T* the temperature.

At 300 K, the *χ*_M_*T* value of 28.0 cm^3^ mol^−1^ K for **1** is lower than the value of 29.5 cm^3^ mol^−1^ K, expected for spin-only contributions to the magnetism, assuming *g*=2.0 for both Mn^II^ and Mn^III^, *g* being the isotropic *g* value. On lowering the temperature, the *χ*_M_*T* product remains essentially constant down to a temperature of approximately 150 K where it begins to decrease rapidly to reach a minimum value of 10.8 cm^3^ mol^−1^ K at 5 K. The spin-Hamiltonian matrix of **1** is a square matrix of dimension 810,000 and cannot be diagonalised by standard techniques. Thus, to model the *χ*_M_*T* product, we have employed home-written software (ITO-MAGFIT)[18] that makes use of irreducible tensor operator algebra[19] to block-diagonalise the spin-Hamiltonian matrix. ITO-MAGFIT is a magnetisation fitting program that uses the Levenberg–Marquardt algorithm.[20] We used the general form of the isotropic spin-Hamiltonian of Equation (1) to model the *χ*_M_*T* product:


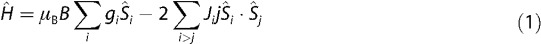
(1)

where *μ*_B_ is the Bohr magneton, the index *i* runs through all constitutive single-ion centres, *g* is the *g* matrix of the *i* th single-ion, here assumed to be *g*=2.0 for simplicity, *Ŝ* is a single-ion spin operator, and *J* is the isotropic exchange parameter between a pair of metal centres. To avoid over-parameterisation we have assumed that all Mn^II^–Mn^II^, all Mn^II^–Mn^III^ and Mn^III^–Mn^III^ isotropic exchange parameters are the same, This results in three independent fit parameters to account for the pairwise isotropic exchange interaction terms: *J*_1–2_, *J*_1–3_, *J*_2–4_, *J*_3–4_, *J*_3–5_, *J*_4—7_, *J*_5–6_, *J*_5–7_, *J*_6–8_, *J*_7–8_ between Mn^II^ and Mn^III^ centres (hereafter referred to as *J*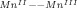); *J*_2–3_ and *J*_6–7_ between Mn^II^ centres (*J*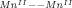); and *J*_4–5_ between Mn^III^ centres (*J*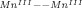). These pairwise isotropic exchange parameters are schematically represented in the inserts of Figure 5. Under these conditions, the best-fit parameters for **1** are: *J*
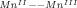
=+0.92 cm^−1^, *J*
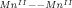
=−4.48 cm^−1^ and *J*
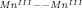
=−1.52 cm^−1^. The best-fit curve is shown in Figure 5. These values are in good agreement with the parameters previously determined for molecules containing the same inverted butterfly topology of Mn^II^ and Mn^III^ centres.[4, 5, 21] With these parameters, the ground spin-state of **1** is a singlet (*S*=0), with numerous excited spin-states lying in very close proximity (Figure S11 in the Supporting Information).

At 300 K, the *χ*_M_*T* value of 38.0 cm^3^ mol^−1^ K for **2** is slightly higher than the value of 36.5 cm^3^ mol^−1^ K, expected for spin-only contributions to the magnetism of **2**, assuming *g*=2.0 for Mn^II^, Mn^III^ and Gd^III^. On lowering the temperature, the *χ*_M_*T* product of **2** remains constant down to *T*=150 K wherefrom it begins to increase, reaching a maximum value of 53.2 cm^3^ mol^−1^ K at 5 K. The spin-Hamiltonian matrix of **2** is a square matrix of dimension 1,440,000 and, as with **1**, cannot be diagonalised by standard matrix diagonalisation techniques.

Following the same strategy as employed for the interpretation of the *χ*_M_*T* product of **1**, we again chose to include the minimum number of fit parameters. Thus, we fixed the value of isotropic exchange parameters *J*_1–2_, *J*_2–4_, *J*_5–7_, *J*_7–8_, taking into account the exchange between Mn^II^ and Mn^III^ centres (*J*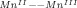) to the best-fit value (+0.92 cm^−1^) of the equivalent parameter obtained for **1**, and used as free fit parameters *J*_1–3_, *J*_3–4_, *J*_5–6_, *J*_6–8_, *J*_3–5_, *J*_4–6_ for the exchange between Mn^III^ and Gd^III^ (*J*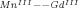), *J*_2–3_ and *J*_6–7_, for the exchange between Mn^II^ and Gd^III^ (*J*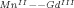), and the *J*_36_ for the exchange between Gd^III^ centres (*J*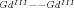). These pairwise isotropic exchange parameters are schematically represented in the relevant insert of Figure 5. Under these conditions, the best-fit parameters for **2** are: *J*
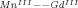
=−0.062 cm^−1^, *J*
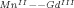
=0.066 cm^−1^ and *J*
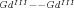
=−0.061 cm^−1^. However, these three best-fit parameters are highly correlated. Thus, their individual determination is impossible from this data set. However, their small magnitude is in agreement with that expected for TM–LnM and LnM–LnM exchange interactions. The best-fit curve is shown in Figure 5. One can see that the determined parameter-set reproduces the experimental data rather well. With these parameters, the ground spin-state of **2** is an *S*=9 state, with numerous excited spin-states lying in close proximity (Figure S12 in the Supporting Information).

**Figure 6:**
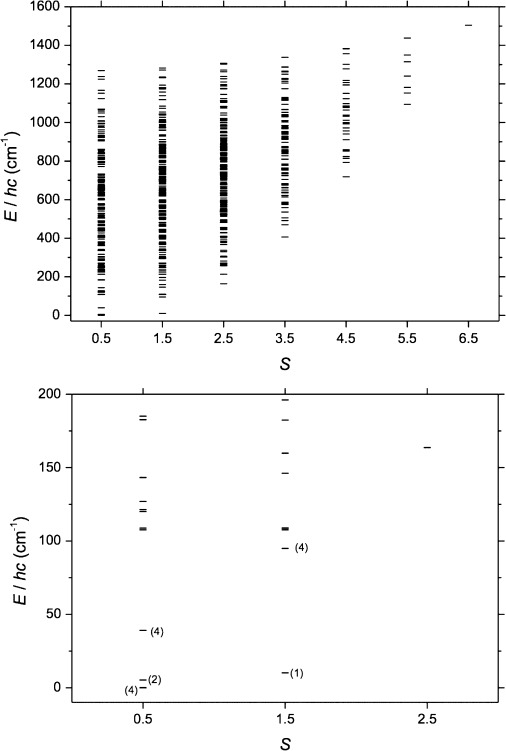
Energy spectrum of 3 determined as described in the main text. Top: full energy spectrum. Bottom: low-lying energy spectrum, with labels denoting the degeneracy of the states.

At 300 K, the *χ*_M_*T* value of 3.1 cm^3^ mol^−1^ K for **3** is significantly lower than the value of 5.9 cm^3^ mol^−1^ K, expected for spin-only contributions to the magnetism of **3**, assuming *g*=2.2 for all Cu^II^ centres. This is indicative of strong antiferromagnetic interactions operating in **3**. On lowering the temperature, the *χ*_M_*T* product monotonically decreases, with the exception of a plateau-like region around 25 K, to reach a minimum value of 0.5 cm^3^ mol^−1^ K at 5 K. The spin-Hamiltonian matrix of **3** is a square matrix of dimension 8,192 and is much easier to handle than the spin-Hamiltonian matrices of **1** and **2**. Thus, we measured variable-field and variable-temperature (VTVB) magnetisation data in the temperature range 2 to 7 K and in the magnetic field range 0.5 to 5 T. The variation of the *χ*_M_*T* product and the VTVB data were simultaneously fitted to isotropic spin-Hamiltonian [Eq. (1)], which remains an appropriate model for the magnetic properties of **3**, since single-ion anisotropy terms here are zero (Cu^II^ is *S*=1/2). We chose to neglect antisymmetric interaction terms, often used for the interpretation of the magnetic properties of Cu^II^-containing complexes,[22] since these proved to be unnecessary. Thus, for the interpretation of the magnetic properties of **3** we included three free parameters in spin-Hamiltonian [Eq. (1)] (Figure 5): *J*_23_, *J*_56_, *J*_89_, *J*_1112_ are used to take into account the exchange between Cu^II^ centres bridged by hydroxides (*J*_1_; the vertices of the square prism); *J*_1–2_, *J*_1–3_, *J*_1–11_, *J*_1–12_, *J*_2–4_, *J*_3–4_, *J*_4–5_, *J*_4–6_, *J*_5–7_, *J*_6–7_, *J*_7–8_, *J*_7–9_, *J*_8–10_, *J*_9–10_, *J*_10–11_, *J*_10–12_, are used to take into account the exchange between the Cu^II^ ions around the peripheral Cu_12_ “wheel” (*J*_2_), and finally *J*_2–13_, *J*_3–13_, *J*_5–13_, *J*_6–13_, *J*_8–13_, *J*_9–13_, *J*_11–13_, *J*_12–13_, are used to take into account the exchange between the central Cu^II^ ion and all of its nearest neighbours (*J*_3_). The best-fit parameters for **3** are: *J*_1_=−84.14 cm^−1^, *J*_2_=−65.97 cm^−1^, *J*_3_=−22.69 cm^−1^. With these parameters, the ground spin-state of **3** is a quadruply degenerate *S*=1/2 state, with the first excited state being a doubly degenerate *S*=1/2 state lying approximately 5.2 cm^−1^ higher in energy. The rest of the energy spectrum of **3** also presents a high degree of degeneracy (Figure 6), suggesting a particularly exceptional case of spin-frustration.[23, 24]

## Conclusions

To conclude, three new cluster types with novel and fascinating topologies have been constructed by enhanced ligand design. All three complexes contain metal-binding properties that are entirely consistent with complexes of TBC[4], which itself can be regarded as a versatile ligand for polymetallic cluster construction (Figure 1). The appropriate combination of two (or more) conjoined ligands is an important step towards enhanced control over topology, nuclearity and properties of polymetallic clusters with these building blocks. Indeed the magnetic exchange between the constituent metal ions in complexes **1**–**3** is very much akin to that observed in molecules built form TBC[4]. Given the large library of TBC[4] clusters known, the correlation between structure and magnetism in novel bis-TBC[4] cages can become more targeted. Work continues in the controlled construction of polymetallic clusters with bis-TBC[4], the results of which will be reported in due course.

## Experimental Section

Bis-TBC[4] (H_8_L1) was synthesised according to literature procedure.[13] Crystal data for bisTBC[4]⋅(CHCl_3_)_4_: C_46_H_57_Cl_6_O_4_, *M*_r_=886.62, colourless block, 0.25×0.25×0.20 mm^3^, triclinic, space group *P*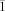 (No. 2), *a*=11.625(2), *b*=13.989(2), *c*=16.333(3) Å, *α*=99.775(7), *b*=103.876(7), *γ*=114.203(7)°, *V*=2241.7(7) Å^3^, *Z*=2, Bruker X8 Apex II CCD diffractometer, Mo_Kα_ radiation, *λ*=0.71073 Å, *T*=100(2) K, 2*q*_max_=48.8°, 23 610 reflections collected, 7333 unique (*R*_int_=0.0594). Final GooF=1.027, *R*1=0.0764, *wR*2=0.1995, *R* indices based on 4206 reflections with *I*>2*σ*(*I*) (refinement on *F*^2^).

### Synthesis of [Mn^III^_4_Mn^II^_4_(L1)_2_(μ_3_-OH)_2_(μ-OH)(μ-Cl)_0.5_(H_2_O)_0.5_ (MeOH)_0.5_(μ-HCO_2_)_0.5_(dmf)_4_]⋅(H_2_O)_2_(MeCN)_12_ (1)

H_8_L1 (200 mg, 0.172 mmol) and MnCl_2_⋅4 H_2_O (68 mg, 0.344 mmol) were suspended in a 1:1 mixture of DMF/MeOH (24 mL). NEt_3_ (0.4 mL) was added and the resulting purple solution was stirred for 90 min and then filtered. The mother liquor was allowed to diffuse slowly with acetonitrile vapour, affording a crop of crystals from which a crystal suitable for X-ray diffraction studies was selected. Yield (101 mg, 30 %); elemental analysis (%) calcd for **1**, C_213_H_277.5_N_16_O_26.5_Cl_0.5_Mn_8_: C 64.88, H 7.09, N 5.68; found: C 65.17, H 7.25, N 5.57. Crystal data for **1**: C_213_H_277.50_Cl_0.50_Mn_8_N_16_O_26.50_, *M*_r_=3943.26, purple block, 0.13×0.11×0.08 mm^3^, orthorhombic, space group *Pccn* (No. 56), *a*=41.2669(15), *b*=31.9796(12), *c*=32.3854(13) Å, *V*=42 739(3) Å^3^, *Z*=8, Bruker APEX II CCD diffractometer, synchrotron radiation, *λ*=0.77490 Å, *T*=100(2) K, 2*θ*_max_=55.9°, 302 992 reflections collected, 39 354 unique (*R*_int_=0.0683). Final GooF=1.700, *R*1=0.0788, *wR*2=0.2362, *R* indices based on 30 436 reflections with *I*>2*σ*(*I*) (refinement on *F*^2^).

### Synthesis of [Mn^III^_4_Mn^II^_2_Gd^III^_2_(L1–8 H)_2_(Cl)_2_(μ_3_-OH)_4_(MeOH)_2_ (dmf)_8_]⋅(Et_2_O)_5_(dmf) (2)

H_8_L1 (200 mg, 0.172 mmol), MnCl_2_⋅4 H_2_O (68 mg, 0.344 mmol) and GdCl_3_⋅6 H_2_O (64 mg, 0.172 mmol) were stirred in a solvent mixture of DMF/MeOH (12 mL:12 mL). NEt_3_ (0.4 mL) was added and the reaction was stirred for 90 min. After filtration, a crop of dark green block crystals, suitable for X-ray diffraction studies, were grown by vapour diffusion of the mother liquor with diethyl ether. Yield (81 mg, 21 %); elemental analysis (%) calcd for **1**, C_225_H_329_Cl_2_Gd_2_Mn_6_N_9_O_36_: C 60.71, H 7.45, N 2.83; found: C 60.30, H 7.14, N 2.95. Crystal data for **2**: C_225_H_329_Cl_2_Gd_2_Mn_6_N_9_O_36_, *M*_r_=4451.01, dark green block, 0.32×0.30×0.25 mm^3^, monoclinic, space group *P*2_1_/*n* (No. 14), *a*=16.731(3), *b*=30.607(6), *c*=21.746(4) Å, *β*=101.89(3)°, *V*=10 896(4) Å^3^, *Z*=2, Bruker X8 Apex II CCD diffractometer, Mo_Kα_ radiation, *λ*=0.71073 Å, *T*=100(2) K, 2*θ*_max_=53.5°, 85 629 reflections collected, 22 772 unique (*R*_int_=0.0697). Final GooF=1.122, *R*1=0.0656, *wR*2=0.1398, *R* indices based on 15 709 reflections with *I*>2*σ*(*I*) (refinement on *F*^2^).

### Synthesis of [Cu^II^_13_(L1)_2_(NO_3_)(μ-OH)_8_(dmf)_7_]⋅(OH)(MeCN)_14_ (3)

H_8_L1 (200 mg, 0.172 mmol) and Cu(NO_3_)_2_⋅3 H_2_O were suspended in a solvent mixture of DMF/MeOH (1 2 mL:12 mL) and stirred for about 10 min. NEt_3_ (0.4 mL) was added and the dark solution was stirred for a further 90 min and then filtered. Brown crystals suitable for single-crystal X-ray diffraction studies were grown by vapour diffusion of the mother liquor with acetonitrile. Yield (108 mg, 27 %); elemental analysis (%) calcd for **1**, C_225_H_304_N_22_O_35_Cu_13_: C 57.46, H 6.52, N 6.55; found: C 57.16, H 6.60, N 6.81. Crystal data for **3**: C_211_H_283_Cu_13_N_15_O_35_, *M*_r_=4605.08, brown needle, 0.10×0.10×0.02 mm^3^, triclinic, space group *P*$\bar 1$
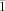
(No. 2), *a*=17.7475(6), *b*=18.0586(6), *c*=20.5598(7) Å, *α*=90.487(2), *β*=111.201(2), *γ*=111.033(2)°, *V*=5661.1(3) Å^3^, *Z*=1, Bruker APEX II CCD diffractometer, synchrotron radiation, *λ*=0.77490 Å, *T*=100(2) K, 2*θ*_max_=67.4°, 88 572 reflections collected, 34 420 unique (*R*_int_=0.0482). Final GooF=1.028, *R*1=0.1425, *wR*2=0.3827, *R* indices based on 26 828 reflections with *I*>2*σ*(*I*) (refinement on *F*^2^).

CCDC-1026245 http://www.ccdc.cam.ac.uk/cgi-bin/catreq.cgi(bisTBC[4]⋅(CHCl_3_)_4_), -1026242 http://www.ccdc.cam.ac.uk/cgi-bin/catreq.cgi(**1**) and -1026243 http://www.ccdc.cam.ac.uk/cgi-bin/catreq.cgi(**2**) and -1026244 http://www.ccdc.cam.ac.uk/cgi-bin/catreq.cgi(**3**) contain the supplementary crystallographic data for this paper. These data can be obtained free of charge from The Cambridge Crystallographic Data Centre via http://www.ccdc.cam.ac.uk/data_request/cif.

## Supporting Information

As a service to our authors and readers, this journal provides supporting information supplied by the authors. Such materials are peer reviewed and may be re-organized for online delivery, but are not copy-edited or typeset. Technical support issues arising from supporting information (other than missing files) should be addressed to the authors.

miscellaneous_information
