# Supplementary material for: Linked Supramolecular Building Blocks for Enhanced Cluster Formation
Source: Chemistry. 2015 Jan 9;21(7):2804–12. doi: 10.1002/chem.201405746 (PMC4517171; doi:10.1002/chem.201405746)
Supplement: Supplementary file 1 — miscellaneous_information [file chem0021-2804-sd1.pdf]

CHEMISTRY
A European Journal

Supporting Information

© Copyright Wiley-VCH Verlag GmbH & Co. KGaA, 69451 Weinheim, 2015

Linked Supramolecular Building Blocks for Enhanced Cluster Formation

Ross McLellan,[a] Maria A. Palacios,[b] Christine M. Beavers,[c] Simon J. Teat,[c] Stergios Piligkos,*[d] Euan K. Brechin,*[b] and Scott J. Dalgarno*[a]

chem_201405746_sm_miscellaneous_information.pdf

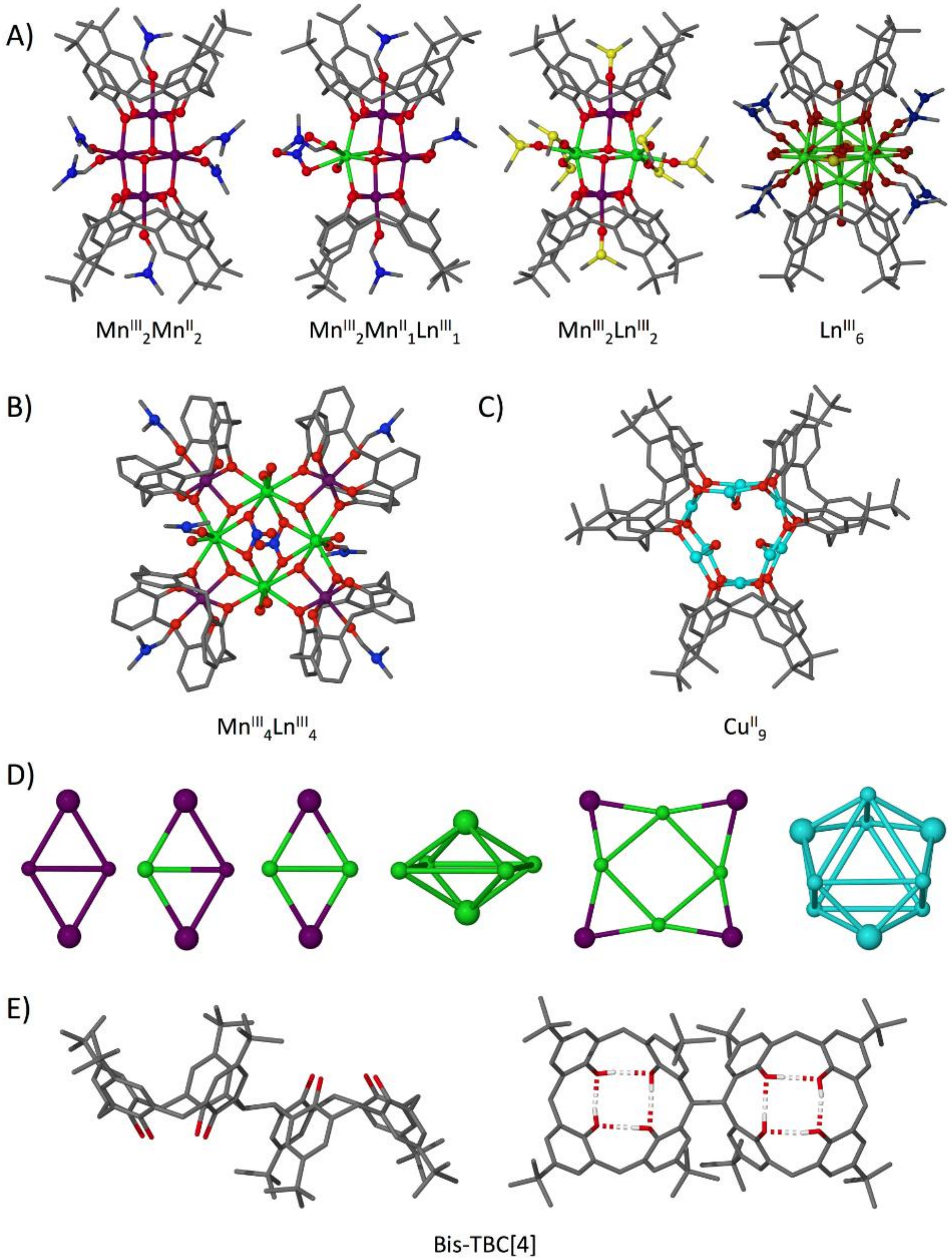

Figure 1. Large version of Figure 1 in manusript.

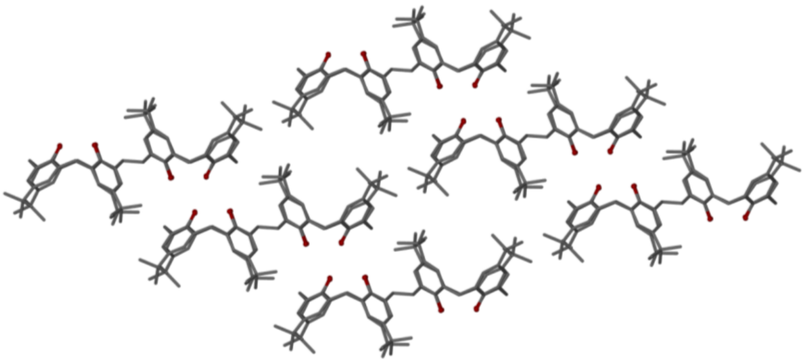

Figure S1 Packing diagram of H8L1, co-crystallised solvent and hydrogen atoms omited for clarity.

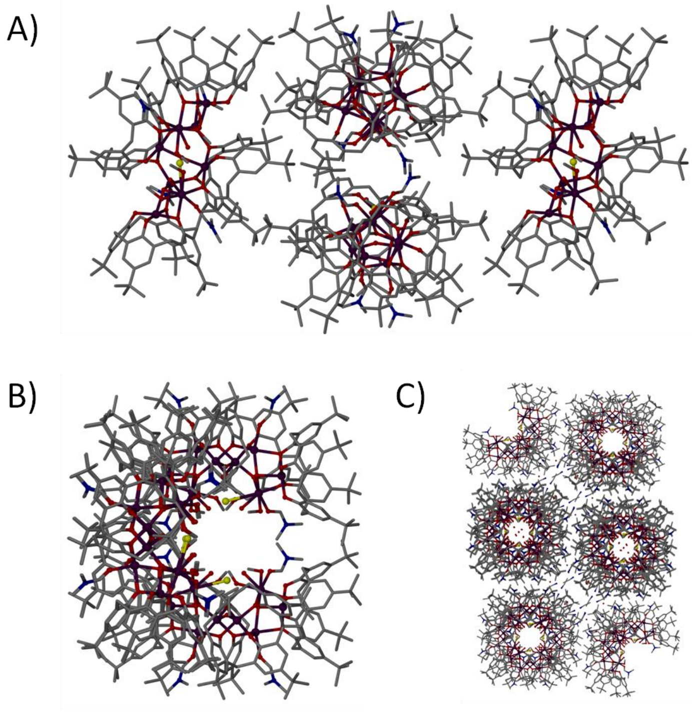

Figure S2 A) Packing of (one component of disordered) **1** showing perpendicular arrangement of molecules. B) View of A) from a perpendicular direction. C) Extended packing of **1** showing tube-like assemblies.

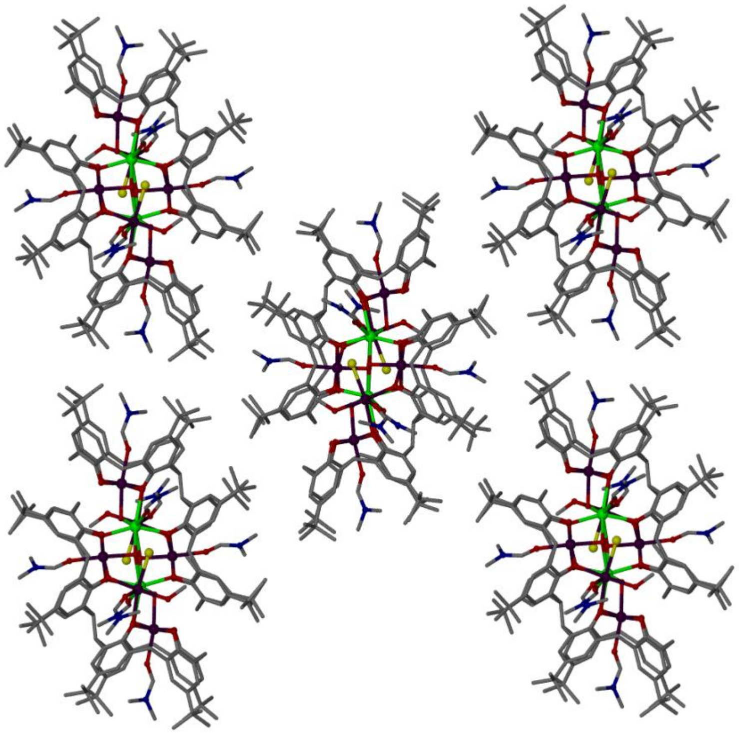

Figure S3 Packing diagram of **2** showing head-to-head zig-zag arrangement.

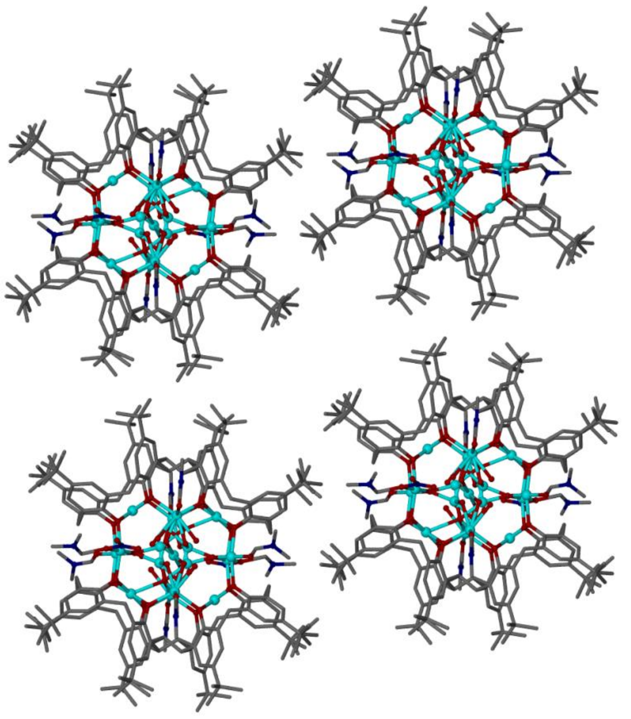

Figure S4 Packing diagram of **3** showing offset linear chain arrangement.

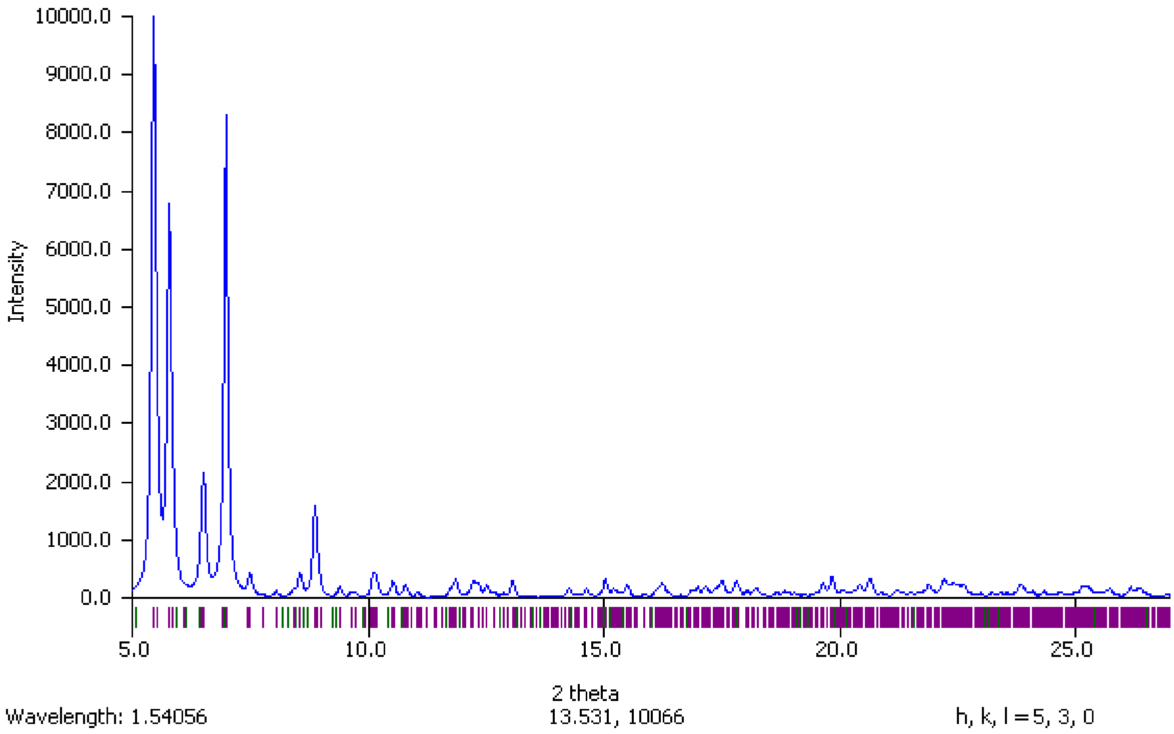

Figure S5 Calculated PXRD pattern of **1**.

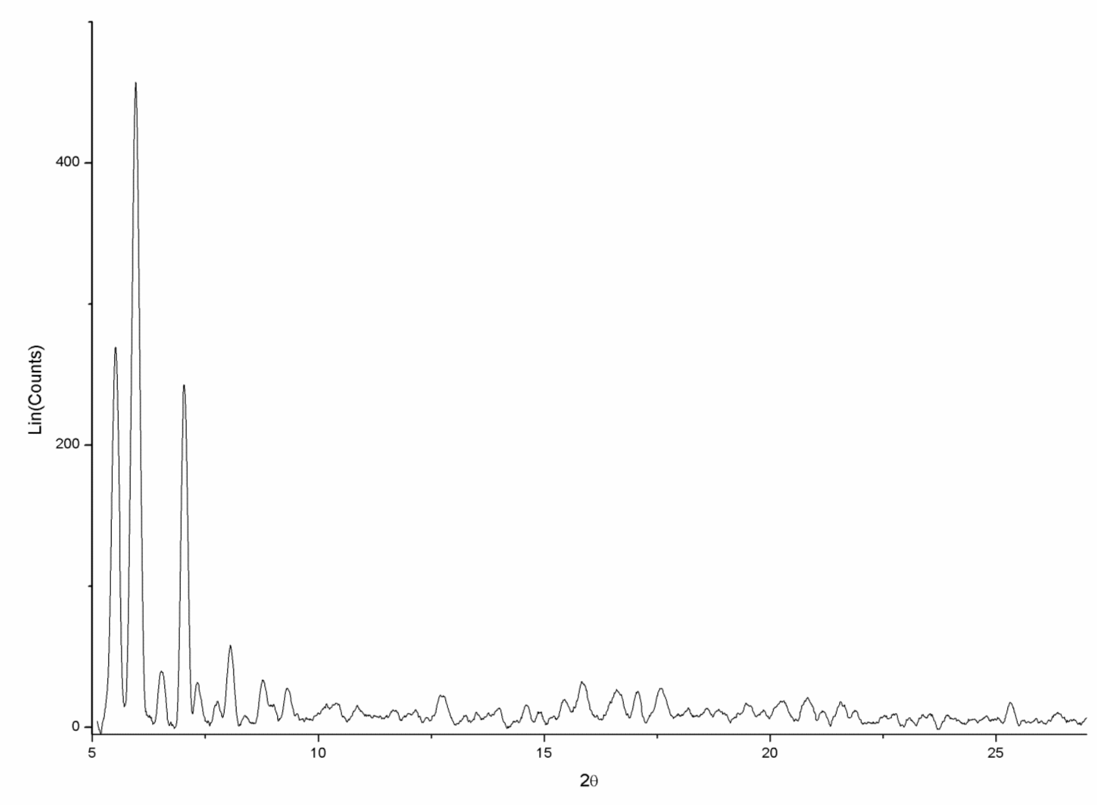

Figure S6 PXRD pattern of **1**.

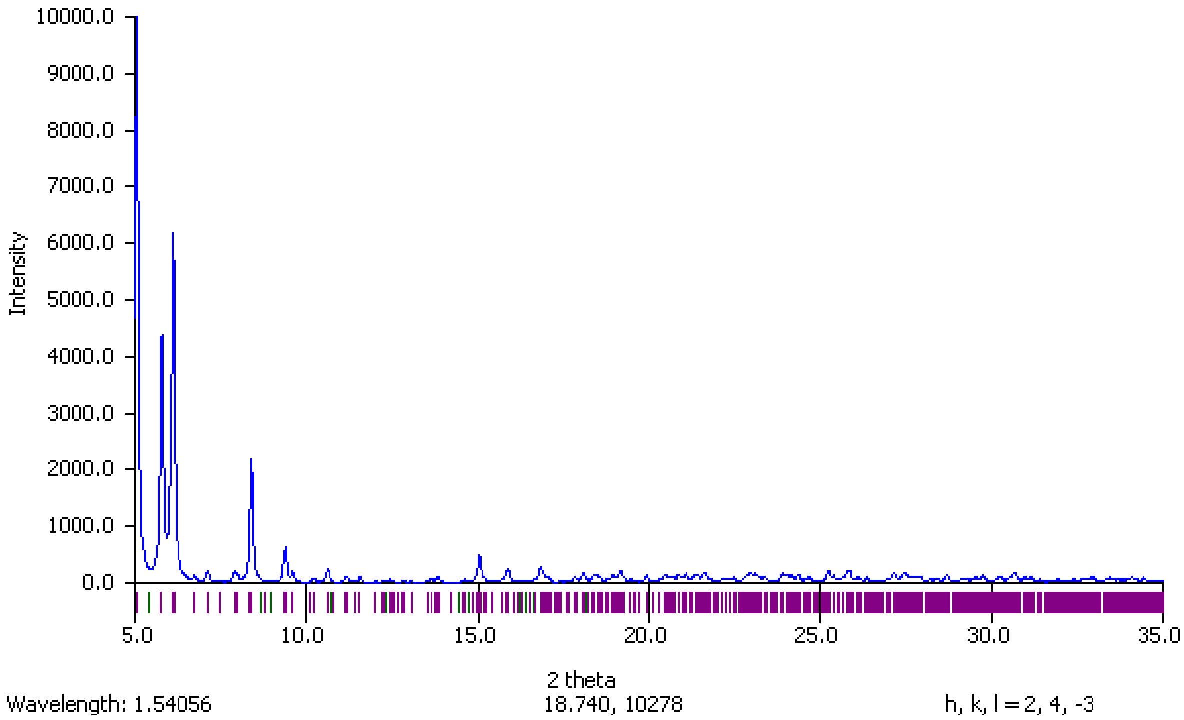

Figure S7 Calculated PXRD pattern of **2**.

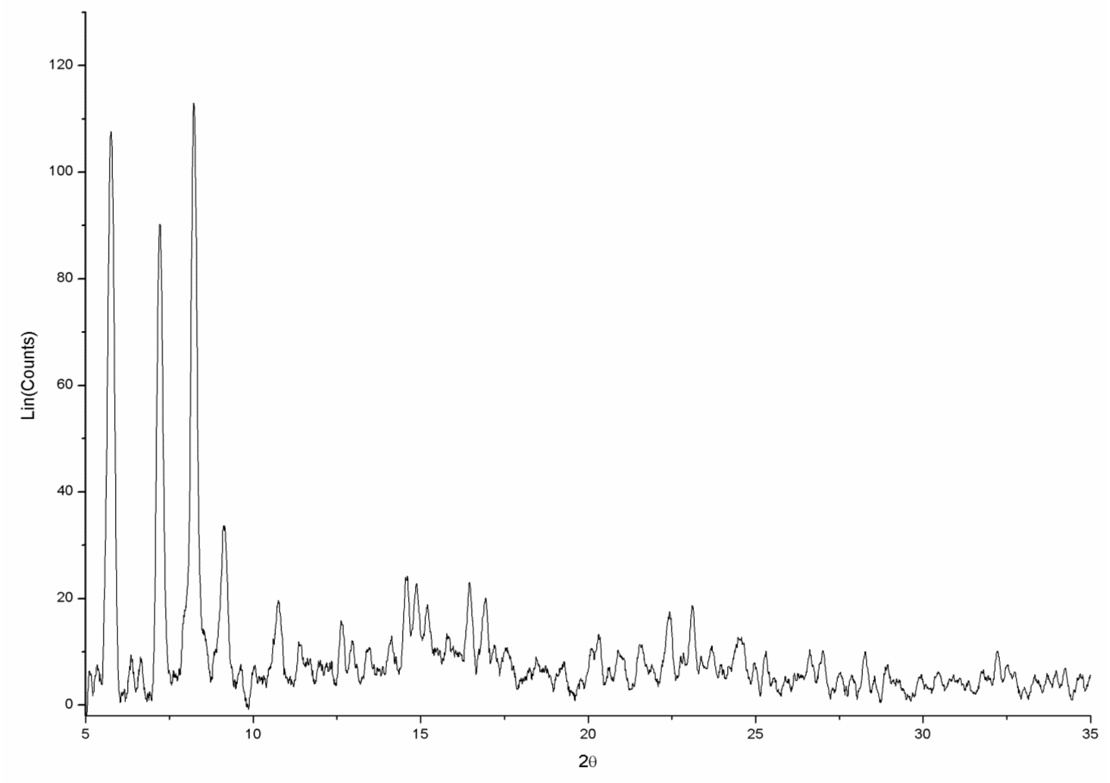

Figure S8 PXRD pattern of **2**.

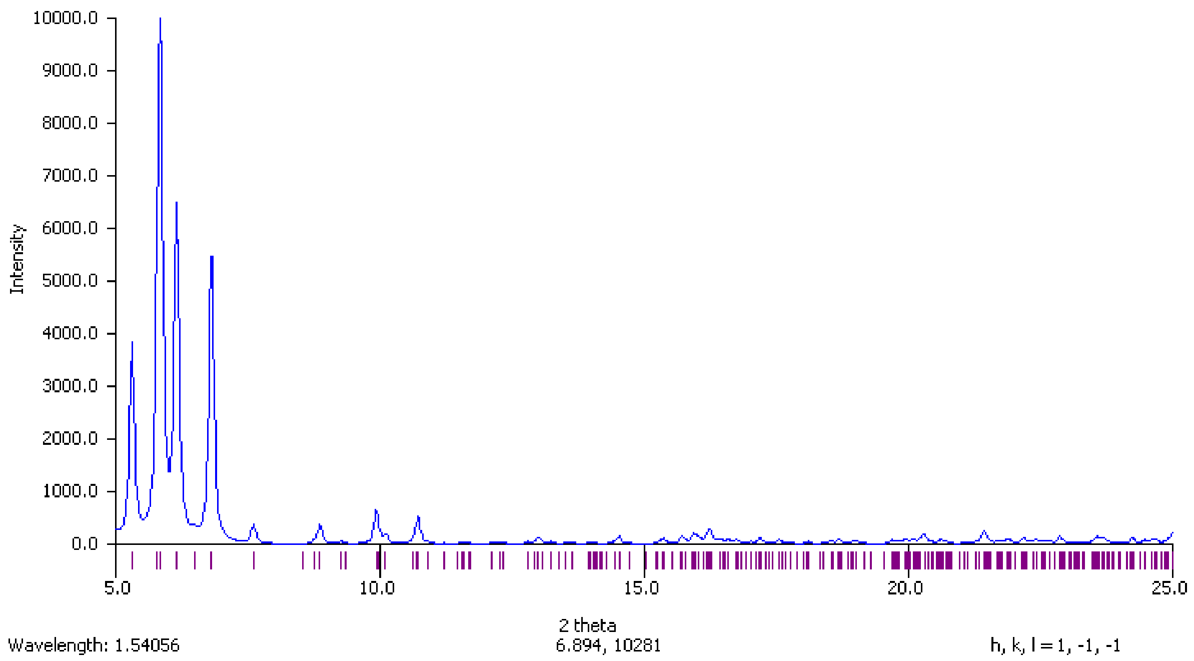

Figure S9 Calculated PXRD pattern of **3**.

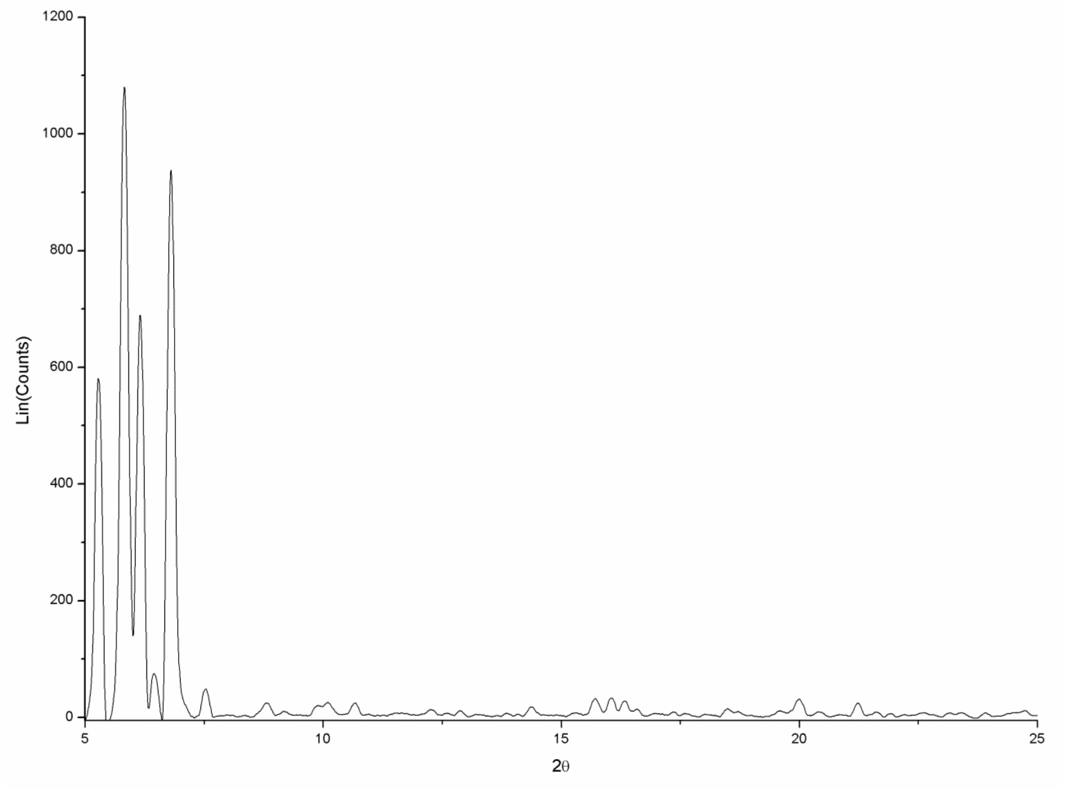

Figure S10 PXRD pattern of **3**.

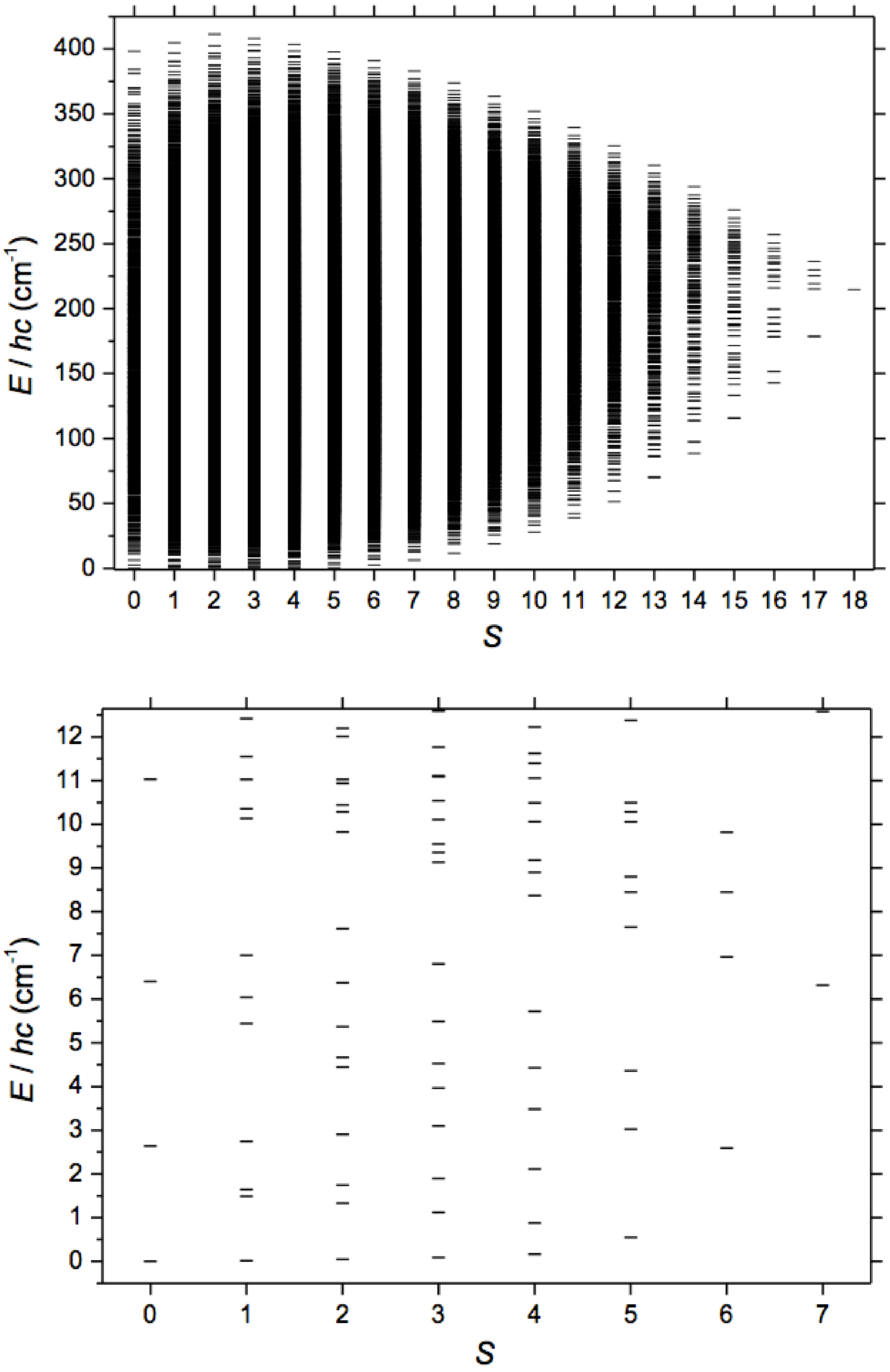

Figure S11. Energy spectrum of **1** determined as described in the main text. Top: full energy spectrum. Bottom: Low-lying energy spectrum.

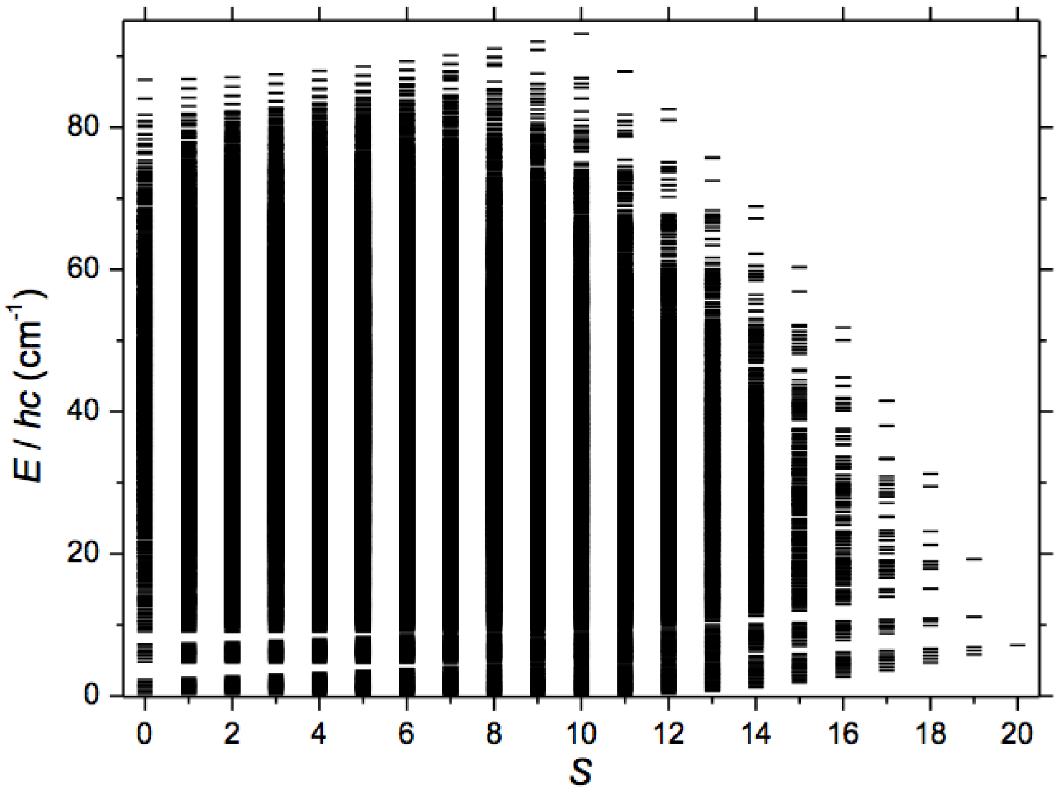

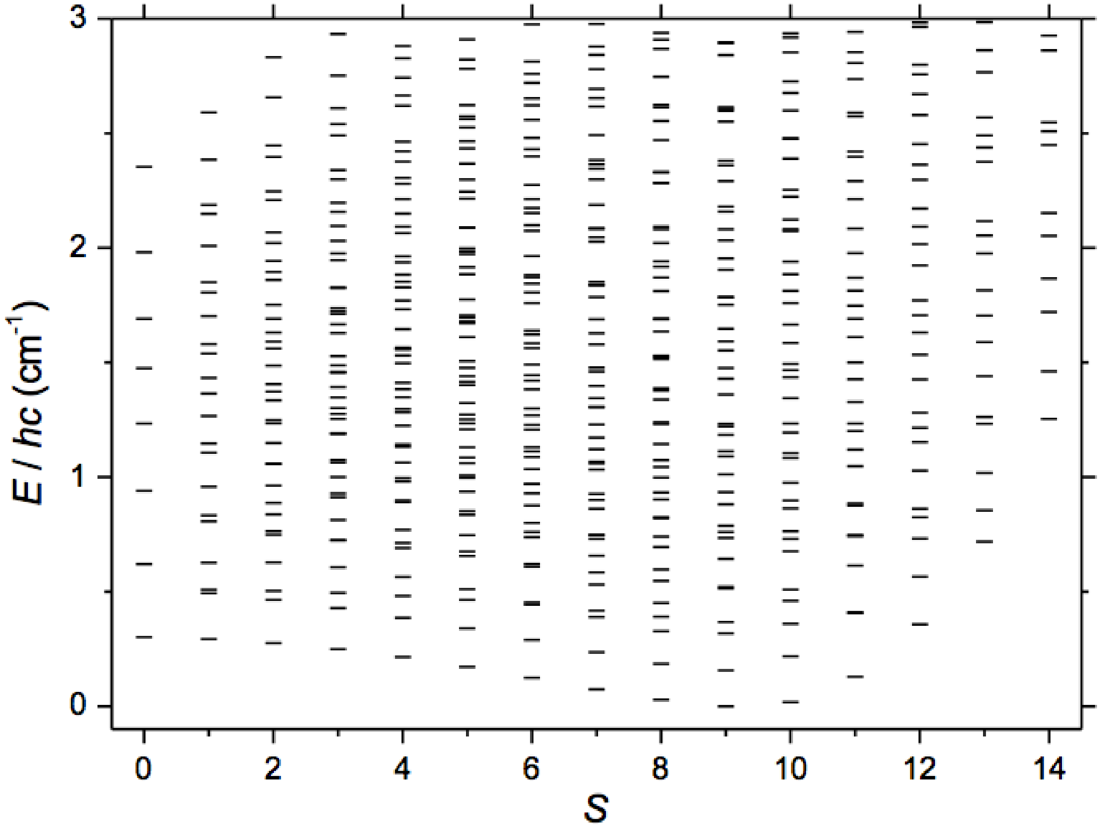

Figure S12. Energy spectrum of **2** determined as described in the main text. Top: full energy spectrum. Bottom: Low-lying energy spectrum.
